# Physiological Heterogeneity Triggers Sibling Conflict Mediated by the Type VI Secretion System in an Aggregative Multicellular Bacterium

**DOI:** 10.1128/mBio.01645-17

**Published:** 2018-02-06

**Authors:** Vera Troselj, Anke Treuner-Lange, Lotte Søgaard-Andersen, Daniel Wall

**Affiliations:** a Department of Molecular Biology, University of Wyoming, Laramie, Wyoming, USA; b Department of Ecophysiology, Max Planck Institute for Terrestrial Microbiology, Marburg, Germany; University of Washington; University of Washington

**Keywords:** kin discrimination, conflict, cooperation, myxobacteria, self-recognition, type VI secretion system

## Abstract

A hallmark of social microorganisms is their ability to engage in complex and coordinated behaviors that depend on cooperative and synchronized actions among many cells. For instance, myxobacteria use an aggregation strategy to form multicellular, spore-filled fruiting bodies in response to starvation. One barrier to the synchronization process is physiological heterogeneity within clonal populations. How myxobacteria cope with these physiological differences is poorly understood. Here, we investigated the interactions between closely related but physiologically distinct *Myxococcus xanthus* populations. We used a genetic approach to create amino acid auxotrophs and tested how they interact with a parental prototroph strain. Importantly, we found that auxotrophs were killed by their prototroph siblings when the former were starved for amino acids but not when grown on rich medium or when both strains were starved. This antagonism depended on the type VI secretion system (T6SS) as well as gliding motility; in particular, we identified the effector-immunity pair (TsxEI) as the mediator of this killing. This sibling antagonism resulted from lower levels of the TsxI immunity protein in the starved population. Thus, when starving auxotrophs were mixed with nonstarving prototrophs, the auxotrophs were susceptible to intoxication by the TsxE effector delivered by the T6SS from the prototrophs. Furthermore, our results suggested that homogeneously starving populations have reduced T6SS activity and, therefore, do not antagonize each other. We conclude that heterogeneous populations of *M. xanthus* use T6SS-dependent killing to eliminate starving or less-fit cells, thus facilitating the attainment of homeostasis within a population and the synchronization of behaviors.

## IMPORTANCE

Social bacteria employ elaborate strategies to adapt to environmental challenges. One means to prepare for unpredictable changes is for clonal populations to contain individuals with diverse physiological states. These subpopulations will differentially respond to new environmental conditions, ensuring that some cells will better adapt. However, for social bacteria physiological heterogeneity may impede the ability of a clonal population to synchronize their behaviors. By using a highly cooperative and synchronizable model organism, *M. xanthus*, we asked how physiological differences between interacting siblings impacted their collective behaviors. Physiological heterogeneity was experimentally designed such that one population starved while the other grew when mixed. We found that these differences led to social conflict where more-fit individuals killed their less-fit siblings. For the first time, we report that the T6SS nanoweapon mediates antagonism between siblings, resulting in myxobacterial populations becoming more synchronized to conduct social behaviors.

## INTRODUCTION

Multicellular organisms can arise by two fundamental strategies. In one case, multicellularity develops from a single cell, often a fertilized egg. Here, a single cell serves as a bottleneck through which multicellularity arises by clonal expansion. This ensures that all progeny cells are genetically identical and physiologically similar, at least within tissues, as cell growth occurs in the protective environment of the organism. Alternatively, multicellularity occurs by the aggregation of cells from the environment. Here, cells may have genetic, adaptive, age, and/or fitness differences. These differences lead to heterogeneous cell populations that may impede the cooperative interactions among cells required for multicellularity. Indeed, organisms that evolved multicellularity by an aggregative strategy develop into organisms with limited complexities, namely, fruiting bodies, as opposed to the more complex cell types and tissues associated with plant and animal species (1).

*Myxococcus xanthus* is a model bacterium that forms multicellular fruiting bodies by the aggregation of thousands of cells in response to starvation. During vegetative growth, *M. xanthus* lives in the soil as a microbial predator. Through gliding motility, *M. xanthus* cells explore their environment for food as social packs or as individuals. Their social behaviors require intercellular communication, motility, and an ability to synchronize the actions of thousands of cells to build spore-filled fruiting bodies (2). Engaging in social interactions is energetically costly for participant cells in terms of signal production, resource sharing, coordinating behaviors, and the differentiation of specialized cell types in which all cells do not necessarily share equal benefits (3). In addition, cooperative behaviors lead to conditions that can be exploited by individuals that use shared resources but do not equally contribute to their production. Therefore, mechanisms are needed to regulate social interactions and to limit exploitation by so-called cheater cells (4).

To address genetic heterogeneity and possible social conflicts, aggregative species need a way to recognize kin and/or discriminate against nonkin. Kin recognition in myxobacteria is mediated, in part, by a polymorphic surface receptor called TraA (5–7). Cells that share identical or nearly identical TraA receptors recognize and bind each other and exchange outer membrane proteins and lipids in a process called outer membrane exchange (OME). The consequences of OME depend on the nature of the cargo transferred between partnering cells. In some cases, OME rescues fitness defects associated with cells that contain damaged or missing cell components, such as proteins or lipids (8–10). In other cases, OME delivers toxin effectors, and recipient cells that do not contain cognate antitoxins are discriminated against (11, 12). More broadly, many bacterial species use polymorphic toxins or bacteriocins to eliminate neighboring or competing cells that are not true clonemates. For example, the type VI secretion system (T6SS) is a commonly used system to deliver toxins to adjacent cells. These effectors are delivered through the contraction of an extended sheath that is anchored to the cell envelope and wrapped around an inner tube made up of the Hcp protein. The tip of the tube is composed of a VgrG trimer and PAAR domain proteins. Upon contraction, the T6SS tube penetrates the membrane(s) of target cells and intoxicates them if they lack the cognate antitoxin or immunity protein (13, 14).

Much less is known about how bacteria deal with physiological differences among cells within a clonal population and how such heterogeneity influences cell-cell interactions. Even in genetically uniform communities, physiological differences emerge because of spatial variations in nutrient availability and physical and/or chemical differences within microenvironments (15). In addition, cells vary by age, the damage load that they carry (e.g., their level of oxidized proteins and extent of membrane oxidation), and adaptation differences, along with stochastic differences in gene expression or phase variation (16–18). Whereas physiological heterogeneity may offer fitness benefits to populations by providing a wider array of adaptive responses to changing environments (19–21), the ability of social microbes to coordinate behaviors largely depends on synchronized actions of cells responding collectively to cellular and environmental cues. Because cells within populations are physiologically different, we hypothesized that *M. xanthus* evolved a mechanism(s) to manage heterogeneity, thus facilitating a transition toward the synchronization of social behaviors required for multicellularity.

Here, we examined how genetic siblings interact within physiologically distinct *M. xanthus* populations. To address this, we created auxotroph strains and mixed them with their parental prototroph strain on minimal medium. In these mixtures, one population starves while the other grows. We asked whether these kin cells antagonize, cooperate with, or act indifferently toward one another. We found that antagonism occurred between kin *M. xanthus* strains only under conditions under which they were physiologically distinct. We show that this antagonism is dependent on the T6SS, and we identify the effector-immunity pair that mediates this interaction, which provides a model for how antagonism occurs.

## RESULTS

### Phenotypic analysis of auxotroph mutants.

**FIG 1:**
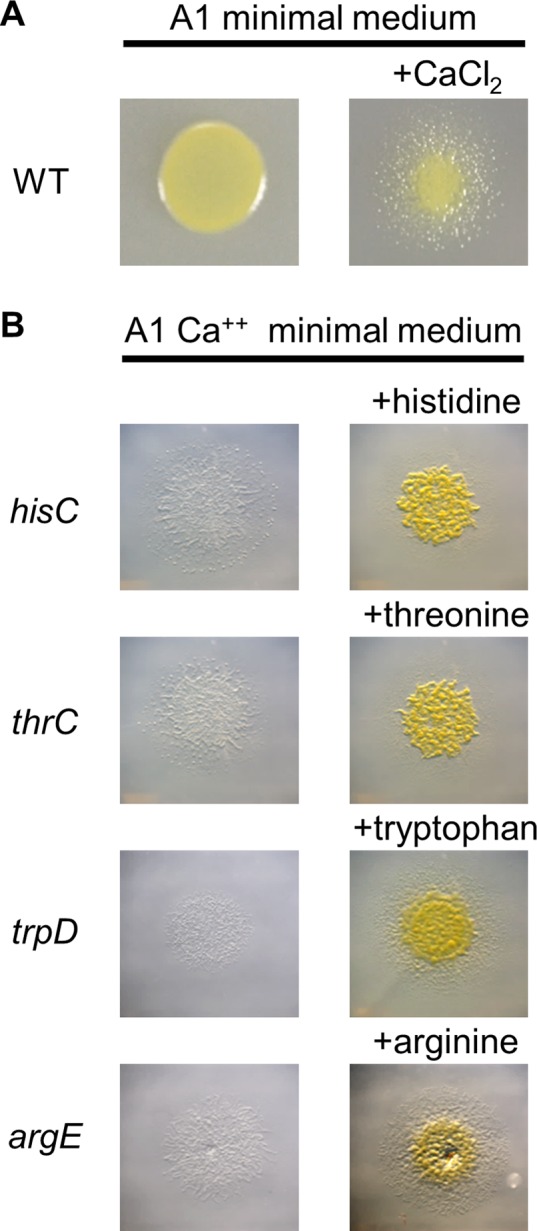
Phenotypic properties of strains on minimal medium. (A) Cell motility in DK1622 (WT) is blocked on A1 and is restored by addition of 2 mM CaCl_2_ to A1 (A1 Ca^2+^). (B) Gene disruptions were made in ORFs predicted to be involved in amino acid biosynthesis, and the resulting mutants were assessed on A1 Ca^2+^ minimal medium agar (left) and A1 Ca^2+^ supplemented with 100 mg/liter of the indicated amino acids (right). Stereoscope micrographs were taken after a 96-h incubation. Strain details are provided in Table 1.

To test for social interactions between starving and nonstarving populations of kin *M. xanthus* cells, we created auxotroph strains. Auxotrophs were made by disrupting genes predicted to be essential for the biosynthesis of histidine, arginine, threonine, or tryptophan. An auxotroph phenotype of each mutant was tested by its inability to grow on defined A1 minimal medium and the rescue of the growth defect when the medium was supplemented with the corresponding amino acid. However, while conducting these studies, we found that it was necessary to add calcium chloride to restore cell motility on A1; the absence of motility was caused by insufficient Ca^2+^ levels from the ultrapure water and the agar that we used to make A1 (Fig. 1A). Restoring motility was important considering its essential role in a number of *M. xanthus* social behaviors such as predation, development, and OME (22–24). All of the constructed auxotroph mutants exhibited the expected growth phenotypes on A1 (Fig. 1B).

### Auxotroph mutants are conditionally antagonized by a prototroph during mixed growth.

**FIG 2:**
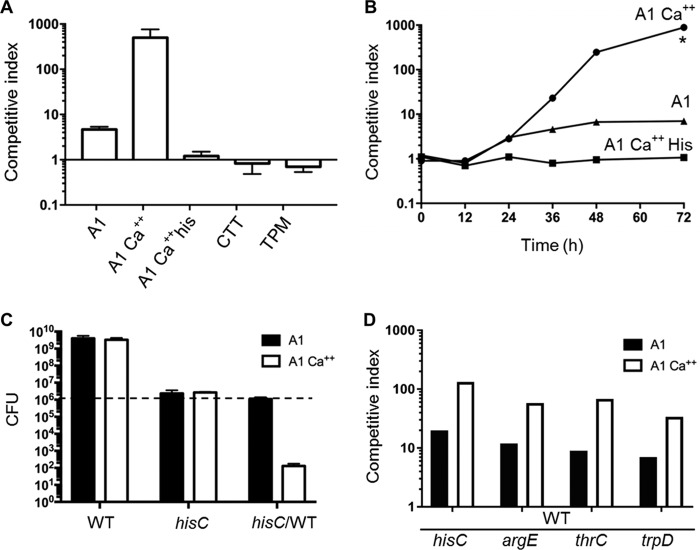
The HisC auxotroph is conditionally antagonized by a prototroph on minimal medium. (A) HisC auxotroph labeled with tdTomato (DW2603) was mixed at a 1:1 ratio with an unlabeled prototroph (WT; DK1622) and plated on the indicated media. After incubation for 48 h, cells were harvested and examined with a microscope to determine the competitive index (prototroph-to-auxotroph ratio). (B) Similar to panel A, except that the competitive index is shown as a function of time on different media. *, no HisC auxotroph cells were detected at this time. (C) A HisC auxotroph (DW2609) and prototroph (DW2412) were plated as monocultures or a mixed culture (*hisC*/WT) on indicated media, and CFU were determined at 72 h. The dashed line indicates CFU for each culture at 0 h. From the mixed culture, only the *hisC* CFU (Km^r^) is shown. All assays, unless stated otherwise, were done in biological triplicates; error bars indicate the SD. (D) Indicated auxotrophs (unlabeled) were mixed with WT labeled with tdTomato (DW2412) at 1:1 starting ratios on the indicated media and incubated for 48 h.

To investigate social interactions between physiologically distinct populations, a histidine auxotroph (*hisC*) labeled with tdTomato was mixed with an unlabeled prototroph strain at a 1:1 ratio and plated on A1 and A1 supplemented with 2 mM CaCl_2_ (A1 Ca^2+^). Mixed cultures incubated on A1 Ca^2+^ supplemented with histidine, rich medium (CTT), or starvation medium (TPM) were used as controls. Prototroph-auxotroph mixtures were harvested to determine the competitive index, which was expressed as a change in the strain ratio (prototroph to auxotroph) as determined by counting cells under a microscope. After 48 h, mixed colonies from the three control media maintained a near-1:1 ratio. In contrast, the competitive index from minimal media, both A1 and A1 Ca^2+^, was increased in favor of the prototroph (Fig. 2A). The finding that the prototroph cells outcompeted the auxotroph cells was an expected outcome because the growth of the latter strain is impaired on A1 medium lacking histidine (Fig. 1B). However, there was a dramatic difference in strain ratios between A1 and A1 supplemented with calcium. Notably, auxotroph cells were almost undetectable by microscopy examination of mixed colonies derived from A1 Ca^2+^. A time course experiment showed an unchanged strain ratio during the first 12 h, followed by a steady increase in prototroph numbers on A1 that plateaued after 48 h, whereas on A1 Ca^2+^ the change was more dramatic and resulted in the complete depletion of auxotroph cells by 72 h (Fig. 2B). As outlined below, when the tdTomato marker was instead placed in the prototroph strain, the auxotroph was similarly depleted in competition on A1 Ca^2+^, indicating that this marker does not interfere with this assay. Additionally, antagonism measured by CFU, which determines viable cell numbers, showed that the number of auxotroph cells was reduced by 10,000-fold when grown with prototrophs on A1 Ca^2+^, compared with auxotroph grown as a monoculture on the same medium (Fig. 2C). In contrast, there was no difference in the ability of this auxotroph to survive in a mixed culture incubated on A1 compared with a monoculture on the same medium (Fig. 2C). These results suggested that the auxotroph on A1 Ca^2+^ was not merely outcompeted by the prototroph because of nutrient limitation but was also antagonized. Notably, antagonism was absent on A1 and when both strains were able to grow (on A1 Ca^2+^ with histidine and CTT) as well as in the case when both strains were starved on TPM, which does not contain amino acids (Fig. 2A). We note that the difference in the degree of auxotroph depletion as determined by microscopy (competitive index) compared with CFU was likely due to the presence of some nonviable auxotroph cells that were nevertheless scored under the microscope. Despite this quantitative difference, we found the competitive index to be an accurate and reproducible method for detecting antagonism and used it subsequently because of its rapid readout.

To test whether this antagonism was a specific phenotype of the *hisC* mutant, three other auxotroph mutants (*thrC*, *trpD*, and *argE*) were tested. Notably, all three mutants showed the same phenotype as the HisC auxotroph, indicating that a prototroph strain generally antagonizes amino acid auxotroph mutants when they are coincubated on the medium that is nonpermissive for auxotroph growth (Fig. 2D). For simplicity, we continued to use the HisC auxotroph in further experiments.

### Antagonism depends on motility but not OME.

**FIG 3:**
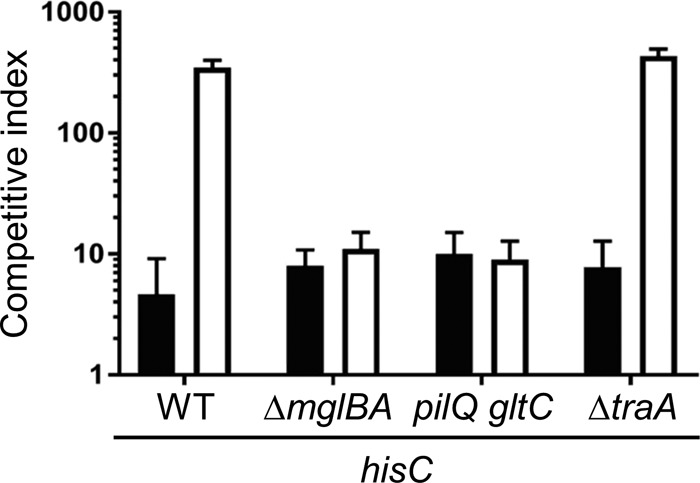
Starvation-induced antagonism depends on motility but not OME. WT (DK1622), Δ*mglBA* nonmotile (DK6204), *pilQ gltC* double mutant (DW2622), and OME mutant (DW1480; Δ*traA*) strains were each mixed with a tdTomato-labeled HisC auxotroph. The competitive index was determined at 48 h as described for Fig. 2A. Black bars, A1 medium; white bars, A1 Ca^2+^ medium.

As described above, we found that antagonism on A1 required Ca^2+^ and asked what role this ion might play. Considering that the only phenotypic difference observed between wild type (WT) incubated with and that incubated without Ca^2+^ was a reduction in motility in the absence of Ca^2+^ (Fig. 1A), we tested whether motility facilitates antagonism. Here, two different nonmotile prototroph mutants, *mglBA* and *pilQ gltC* mutants, were each mixed with the HisC auxotroph on A1 Ca^2+^. In both cases, antagonism was abolished (Fig. 3), suggesting that the prototroph requires motility to antagonize the auxotroph. Furthermore, we found the same outcome when both the prototroph and auxotroph strains were genetically nonmotile and mixed on A1 Ca^2+^ (data not shown), demonstrating that motility plays a critical role in antagonism.

In our previous studies, we showed that OME can lead to sibling antagonism that is mediated by the exchange of toxins between cells (11, 12). To address the possibility that OME is involved in prototroph-auxotroph antagonism, the HisC auxotroph was mixed with a Δ*traA* prototroph that is defective in OME. This Δ*traA* mutant showed comparable antagonism as the WT control (Fig. 3), indicating that the antagonism occurs independently of OME.

### Killing is T6SS dependent.

**FIG 4:**
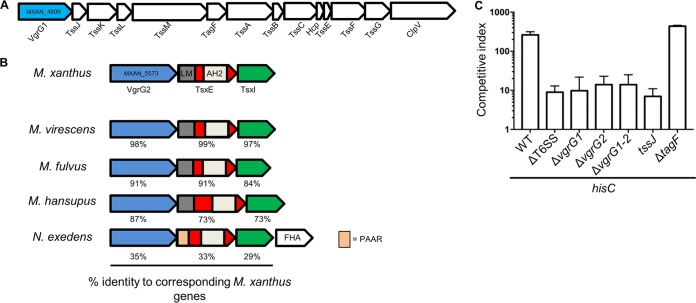
Starvation-induced antagonism is mediated by T6SS. (A) *M. xanthus* T6SS gene cluster (MXAN_4800 to _4013). (B) *M. xanthus vgrG2* (MXAN_5573) gene cluster compared with orthologous clusters found in other myxobacterial species. AH2, amidohydrolase 2 domain; LM, LysM domain; FHA, forkhead-associated domain. (C) Unlabeled WT (control) or T6SS mutants were mixed 1:1 with the HisC auxotroph labeled with tdTomato and plated on A1 Ca^2+^ medium. The competitive index was determined at 48 h.

Considering the magnitude of auxotroph depletion in mixed cultures, we reasoned that antagonism is the consequence of a specific killing mechanism. To investigate this, we conducted competition experiments using prototrophs that contained mutations in predicted or known toxin systems. Included in this analysis were T6SS mutants. *M. xanthus* has 13 core T6SS genes within one gene cluster (Fig. 4A) (25) and an additional orphan *vgrG* gene, *vgrG2*, which shares 43% amino acid sequence identity with VgrG1. Generally, VgrG proteins are structural components of the T6SS located at the tip of the Hcp tube and are essential for effector delivery; alternatively, VgrG proteins can be fused directly to an effector domain to form specialized effectors (26). Whereas other putative toxin system mutants retained the antagonistic phenotype (data not shown), all T6SS mutants, with the exception of a Δ*tagF* mutant, were unable to antagonize the auxotroph (Fig. 4C). TagF is a conserved T6SS protein that functions as a negative posttranslational regulator of T6SS in *Pseudomonas aeruginosa* (27), and our results show it to be nonessential for T6SS activity in *M. xanthus*. This is consistent with the previous finding that the Δ*tagF* mutant still secretes Hcp (28), a process that is a commonly used indicator of T6SS activity (29). Additionally, our findings that both *vgrG* genes were important for antagonism (Fig. 4C) suggest that each *vgrG* gene has a specific, nonredundant function. To eliminate the possibility that the in-frame *vgrG* deletions have polar effects, wild-type versions of these genes were ectopically expressed in each mutant strain. In each case, the *vgrG* killing phenotype was restored, showing that the mutations were complemented and that the *vgrG* in-frame deletions were not polar. We conclude that T6SS mediates antagonism between phenotypically divergent *M. xanthus* strains.

### The VgrG2 operon encodes an effector-immunity pair.

**FIG 5:**
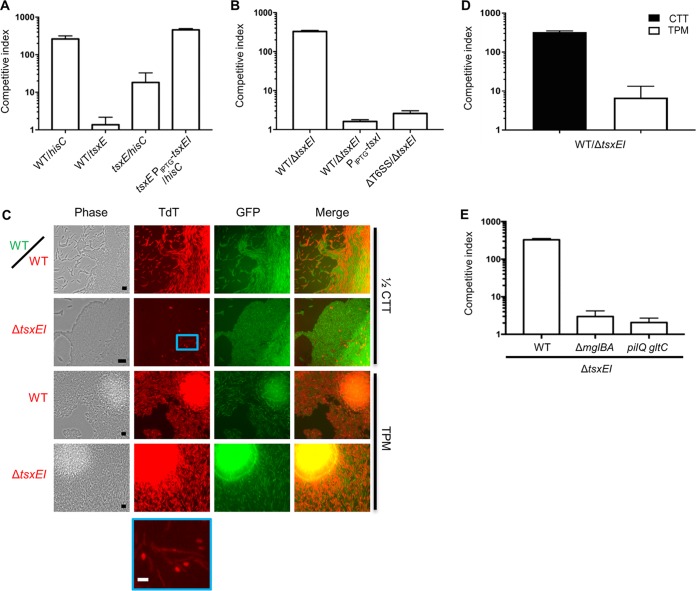
The *vgrG2* gene cluster contains a toxin-immunity gene pair. (A) Unlabeled *tsxE* mutant (DW2650) was mixed with either the WT tdTomato or *hisC* tdTomato strain. The *tsxE* mutation was complemented when the gene was ectopically expressed from an inducible promoter (DW2659; *tsxE* P_IPTG_-*tsxEI*). Strains were plated on A1 Ca^2+^ medium, and the competitive index was determined at 48 h. (B) WT tdTomato was mixed with unlabeled Δ*tsxEI* mutant (DW2655) or this mutant complemented with an inducible *tsxI* gene (DW2667; Δ*tsxEI* P_IPTG_-*tsxI*), and the ΔT6SS tdTomato strain (DW2671) was mixed with a Δ*tsxEI* strain. Strains were plated on CTT, and the competitive index was determined at 48 h. (C) WT VipA-GFP (SA4137) and Δ*tsxEI* tdTomato (TdT) strains were mixed at a 1:1 ratio and plated on the indicated 1% agarose pads. As a control, WT VipA-GFP was mixed with a WT tdTomato strain. Micrographs were taken at 20 h using a 60× objective lens, except for those in the panels second from the top, which were taken using a 100× objective lens, and the blue box area is enlarged at the bottom row to show rounding of Δ*tsxEI* cells. Black bars, 10 μm; white bar, 5 μm. (D) WT tdTomato was mixed with the Δ*tsxEI* mutant and plated on the indicated media, and the competitive index was determined at 72 h. (E) Unlabeled WT or indicated motility mutants (same as in Fig. 3) were mixed with the Δ*tsxEI* tdTomato strain and placed on CTT, and the competitive index was determined at 48 h. All strains were mixed at a 1:1 ratio, and assays were done in triplicate; error bars indicate the SD.

In bacterial species with an orphan *vgrG* gene(s), the open reading frames (ORFs) found immediately downstream sometimes encode T6SS effector-immunity proteins (30). Considering this, we analyzed two ORFs downstream of *M. xanthus vgrG2* (Fig. 4B). By sequence analysis, MXAN_5572, here called TsxE (the putative effector), is predicted to contain amidohydrolase 2 (AH2) and LysM (LM) domains, whereas the protein product of the putative immunity gene MXAN_5571 (TsxI) has no conserved features (31). LysM domains are involved in peptidoglycan binding and are found in many cell wall-degrading enzymes, including some T6SS effectors (32), whereas AH2 belongs to a superfamily of metallo-dependent hydrolases that act on many different substrates (33). This *vgrG2* gene cluster is found in the genomes of several members of the *Myxococcus* genus and in some distantly related *Myxococcales* species such as *Nannocystis exedens* (Fig. 4B). Interestingly, the *N. exedens tsxE* homolog contains a PAAR domain, which along with VgrG composes the tip of the Hcp tube. PAAR domains can also be fused to effector domains or can noncovalently bind T6SS effector cargo (34). To test the possible role of *tsxEI* in the antagonism, we first inactivated *tsxE* in a WT strain. Importantly, this *tsxE* mutant lost its ability to kill the HisC auxotroph on A1 Ca^2+^ medium (Fig. 5A) (note that the *hisC* auxotroph was outcompeted because it cannot grow, but it was not antagonized; compare to Fig. 2A). When mixed with the parent strain (WT), the *tsxE* mutant remained resistant to killing (Fig. 5A). Complementation of the *tsxE* mutant with an ectopic isopropyl-β-d-thiogalactopyranoside (IPTG)-inducible *tsxEI* construct restored the killing phenotype (Fig. 5A). Next, we deleted both the putative effector and immunity gene (Δ*tsxEI*) and conducted competition assays. As expected, this mutant also was unable to kill the auxotroph on solid A1 Ca^2+^ (data not shown). Importantly, the parent strain killed the Δ*tsxEI* mutant even on rich medium (Fig. 5B). Moreover, the parent strain antagonism of the Δ*tsxEI* mutant was abolished by ectopic expression of *tsxI* (Fig. 5B). Direct microscopic examination of the Δ*tsxEI* mutant labeled with tdTomato and mixed with a green fluorescent protein (GFP)-labeled parent strain revealed that the mutant underwent morphological changes whereby the cells became rounded and their numbers decreased (Fig. 5C). In contrast, a ΔT6SS strain did not antagonize the Δ*tsxEI* mutant (Fig. 5B). Together, these results suggest that the T6SS exports toxins from WT cells into neighboring cells, killing the siblings that lack the cognate immunity protein.

It is of note that T6SS-mediated killing was reduced ~100-fold when strain mixtures were placed on TPM starvation agar compared with rich medium (Fig. 5C and D). These results were consistent with our above findings that prototroph-auxotroph antagonism was blocked on TPM starvation agar (Fig. 2A), which suggested to us that starving cells downregulate T6SS function. As the killing was not completely abolished on TPM agar, we speculate that Δ*tsxEI* cells were initially killed by the WT early during coincubation, before the onset of the starvation response and T6SS downregulation. Additionally, killing of Δ*tsxEI* cells was reduced ~100-fold when the WT strains were nonmotile (Fig. 5E), again confirming our above findings that motility plays a key role in the T6SS-mediated attack. Taken together, these results indicate that TsxEI comprise a T6SS-dependent effector-immunity pair.

### TsxI levels decline in starving cells.

**FIG 6:**
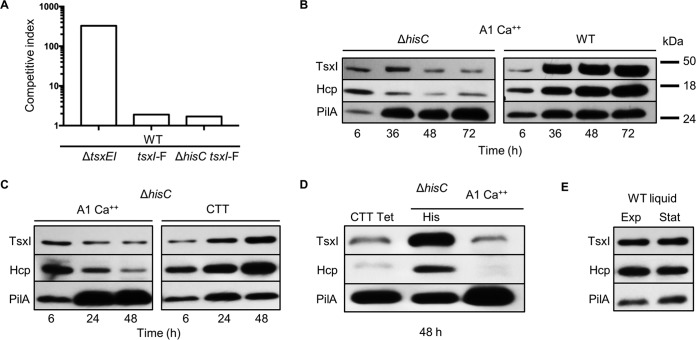
TsxI and Hcp protein levels decrease in starving cells. (A) WT and Δ*hisC* strains expressing TsxI-F (FLAG) (DW2689 and DW2686, respectively) were mixed with the WT strain at a 1:1 ratio on rich medium, and the competitive index was determined at 48 h. (B) Immunoblot assays of WT and a Δ*hisC* strain expressing TsxI-FLAG incubated on A1 Ca^2+^ and harvested at the indicated times. (C) Δ*hisC* mutant incubated on A1 Ca^2+^ or CTT agar. (D) The Δ*hisC* mutant was incubated on CTT with oxytetracycline (CTT Tet), A1 Ca^2+^ His, or A1 Ca^2+^. (E) The WT strain was grown in liquid rich culture medium, and cells were harvested during the exponential phase (Exp) and stationary phase (Stat). All blots were sequentially probed with antibodies to FLAG (TsxI), Hcp, and PilA. Experiments in panels B and C were done in triplicate, the experiment in panel E was done in duplicate, and representative data are shown.

The finding that nutrient-deprived auxotrophs were killed by growing prototroph siblings suggested that TsxI function decreases in the former population. One explanation for this could be that cellular levels of TsxI decrease in response to starvation, thus rendering these cells susceptible to attack by siblings that retain TsxEI. To examine this, we epitope tagged the TsxI immunity protein to generate a TsxI-FLAG protein produced from its native promoter. The TsxI-FLAG fusion was functional as determined by competition assays with the parent strain (Fig. 6A). Next, we compared levels of TsxI in auxotroph and prototroph strain backgrounds when cells were plated separately on A1 Ca^2+^ agar (Fig. 6B). Cells were harvested at various time points, and TsxI-FLAG levels were monitored. In addition to TsxI-FLAG, we also monitored the levels of Hcp, a core component of T6SS (29). As a control, the levels of the major pilin PilA were also assessed. For Western blot analysis, samples were normalized by a Bradford assay to ensure that equal amounts of total protein were loaded in each lane. In the first 6 h after plating on A1 Ca^2+^ medium, there was no obvious difference in TsxI or Hcp levels between the auxotroph and prototroph strains (Fig. 6B). However, between 36 and 72 h the relative amount of TsxI-FLAG and Hcp was much higher in the prototroph than in the auxotroph. As found previously, the relative levels of the PilA control protein increased in the starving cells over time compared with the growing prototroph cells (35). In contrast, the relative levels of TsxI and Hcp increased when the auxotroph was plated on rich CTT medium, compared with when those cells were plated on A1 Ca^2+^ (Fig. 6C). Importantly, adding histidine to A1 Ca^2+^ restored the levels of TsxI-FLAG and Hcp in the HisC auxotroph (Fig. 6D; compare with Fig. 6B). Together, these results suggest that the levels of TsxI-FLAG and Hcp are elevated in growing cells compared with starving cells, in which they decrease over time. Additionally, TsxI and Hcp levels in growing cells increased over time compared to a 6-h time point (Fig. 6B). Because prolonged growth on a surface could alter the growth phase of some cells within a colony (15) and hence alter the relative TsxI and Hcp levels, we tested their relative levels between exponential- and stationary-phase growth in liquid CTT and found no difference (Fig. 6E). This result suggests that growth on a solid surface specifically induces T6SS expression (compare Fig. 6B and E) and is consistent with findings from *P. aeruginosa* (27).

To test if the decreased levels of TsxI-FLAG and Hcp in starved cells are due to these proteins being degraded when growth is blocked, we incubated a tetracycline-sensitive HisC auxotroph on rich medium supplemented with tetracycline, a bacteriostatic antibiotic that inhibits translation. In the presence of a growth-inhibitory concentration of tetracycline, the relative levels of TsxI-FLAG and Hcp decreased, compared to the relative level of PilA (Fig. 6D, left lane), thus recapitulating the results seen in starving cells. These observations imply that under conditions during which protein synthesis is hindered—by amino acid starvation or by blocking translation with an inhibitor—TsxI-FLAG and Hcp proteins are relatively unstable and are degraded, whereas other proteins, such as PilA, are stable, and thus, their relative cellular levels increase. These observations also explain why starving auxotrophic cells are susceptible to T6SS attack when mixed with growing prototrophic cells.

## DISCUSSION

Physiological heterogeneity is generally regarded as a beneficial property of populations, because their overall fitness can increase through a “bet hedging” strategy, whereby phenotypically distinct subpopulations are optimized to quickly adapt to particular changes in the environment to which other subpopulations cannot efficiently respond (21, 36, 37). For example, bacterial populations can contain a subpopulation of dormant persister cells that are able to resist environmental insults, such as exposure to antibiotics, and then those cells can repopulate the biofilm when the insult is removed (20, 38). However, in cases where population fitness depends on cooperative and synchronized cell behaviors, such as for social or aggregative multicellular organisms, population heterogeneity represents a challenge that may hinder the ability of a population of cells to coordinate behaviors (39). To help unify populations for multicellular behaviors, organisms have evolved mechanisms to synchronize their actions. One example is quorum sensing, during which individuals within a population produce and respond to signals made by the population to allow a uniform or coordinated response.

In this study, we show that another form of heterogeneity, involving growing cells mixed with nongrowing siblings, results in antagonism whereby the more-fit growing cells kill their weaker nongrowing siblings. As a consequence, the population moves toward being homogeneous, i.e., all cells are growing, and physiological strife is removed. Since physiological heterogeneity is high within biofilms (15), we hypothesize that the unification of cells’ physiology facilitates coordinated multicellular behaviors in *M. xanthus*.

**FIG 7:**
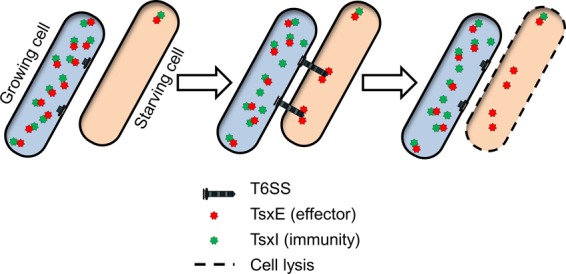
Model for how phenotypic heterogeneity triggers antagonism between sibling cells. The starving cell has reduced TsxI and TsxE levels and is therefore susceptible to TsxE delivery by T6SS. Note that the subcellular localization of TsxE/I is generically depicted, as these proteins may be localized in the periplasm. See the text for further details.

Specifically, we showed that physiological differences in growth rates were policed by T6SS-mediated killing. We further identified an effector-immunity pair (TsxEI) that mediates this antagonism. A Δ*tsxEI* mutant is itself killed when mixed with the parent strain on rich medium, indicating that T6SS function is constitutively active in growing cells. Immunoblot analysis revealed markedly decreased levels of the T6SS protein Hcp as well as of TsxI in starving cells compared with nonstarving growing cells. Based on these findings, we suggest that *M. xanthus* responds to nutrient limitation by decreasing T6SS and Tsx(E)I levels. In response to amino acid starvation, *M. xanthus*, like many bacterial species, induces a stringent response that allows cells to adapt to nutrient depletion (40–42). During the stringent response, cells cannibalize their own proteins to use as an energy source and to provide the building blocks to synthesize an altered proteome and adapt to nutrient limitation. We suggest that TsxI is degraded, along with many other host proteins, during the stringent response, which in turn renders those cells susceptible to attack by TsxE delivered by T6SS from cells that are growing (Fig. 7). In contrast, when the entire population starves, there is a uniform reduction in TsxI levels and T6SS activity, and thus, starving cells are not killed. This scenario provides *M. xanthus* with a mechanism to monitor the growth fitness of adjacent cells; if the population is heterogeneous with respect to growth rates, then less-fit cells are eliminated. Additionally, killing and lysis of siblings may also release nutrients to help those cells that are more fit. Another potential role of this mechanism is to help cells navigate the crucial decision between growth and development under conditions in which nutrients are becoming limiting. Under natural conditions, not all cells may experience nutrient depletion at the same time or to the same extent and will thus respond differently. Cells that, in response to nutrient depletion, enter development prematurely will, as a result, lower their TsxI levels, rendering them vulnerable to T6SS attack by their neighbors. Sibling killing and cannibalism can serve as a way to delay the onset of development as an energetically costly and committed process. Similarly, in *Bacillus subtilis*, Spo0A, a master sporulation regulator, triggers differentiation of matrix-producing cells in biofilms and initiates a cannibalism pathway, mediated by two toxins, Skf and Sdp. Non-matrix-producing cells remain susceptible to intoxication and are lysed and cannibalized by their siblings (43). This cannibalism prolongs vegetative growth and thus delays the onset of sporulation. In the case of myxobacteria, once the perception of starvation is established by the majority of cells, the population can jointly initiate development.

Interestingly, fruiting body development in *M. xanthus* involves death and lysis of more than 50% of the population (44, 45). Programmed cell death has been suggested as the mechanism, although this has not yet been shown and the molecular details are unknown (44, 46). Our data suggest that when cell populations are uniformly starved under laboratory conditions, T6SS does not play a major role in competition because its activity is diminished during development. However, in the wild, environmental and physiological heterogeneity is likely higher, and thus, T6SS could play a role in sibling competition. In separate work, we also showed that the exchange of polymorphic toxins by OME leads to sibling death (11, 12). Future studies are needed to understand if population heterogeneity plays a role in developmental lysis and to determine what molecular mechanism(s) controls this process.

Our experiments demonstrate that killing requires two VgrG proteins with nonredundant functions, where, hypothetically, one VgrG acts as a structural component of the delivery tube and the other acts as a specific adaptor for TsxE delivery. In this scheme, VgrG1 likely serves as a structural component, as its gene is part of the core T6SS gene cluster, whereas *vgrG2* is adjacent to *tsxEI*. Interestingly, other myxobacterial genomes contain many *vgrG* genes (TIGR03361); for example, the *Minicystis rosea* DSM 24000 genome contains 61 paralogs (47). Presumably, a high number of *vgrG* genes allows those organisms to deploy many different toxins by the T6SS. We also found that efficient T6SS-dependent killing in *M. xanthus* requires gliding motility. Because the T6SS apparatus is assembled and is dynamic in nonmotile *M. xanthus* cells (data not shown), motility may facilitate the frequency and precise deployment of T6SS toxins to target cells. In *M. xanthus*, cell motility is also required for other cell-cell contact-dependent processes, such as OME and C-factor signaling during development (23, 24). Since a role for cell motility has not been described for T6SS function in other species, the connection between these processes may be restricted to myxobacteria, which again have many complex social behaviors.

Our work raises a number of questions for future studies. For example, how are T6SS and TsxEI regulated in response to starvation? As the levels of TsxI and Hcp decrease relative to other cellular proteins when *de novo* protein synthesis is blocked by tetracycline, protein turnover likely plays a key role, possibly along with transcriptional control. Because amino acid starvation triggers antagonism, the stringent response could play a role; however, it appears not to be the sole determinant, because we found that a *relA hisC* double mutant was still antagonized on minimal medium (data not shown). Another question concerns the mechanism of action of TsxE. Sequence analysis suggests that TsxE contains a LysM peptidoglycan-binding domain and an amidohydrolase domain, indicating the cell wall as the target. If this is correct, then TsxI would likely localize in the periplasm to neutralize TsxE. The *tsxEI* genes are conserved among several closely related *Myxococcus* species and occasionally also in more distant species such as *N. exedens*. As genes that encode T6SS effectors are not always linked to *vgrG* genes and given the predatory and complex social nature of myxobacteria, there might be other T6SS-dependent effector-immunity pairs encoded by the *M. xanthus* DK1622 genome. Additionally, although TsxEI mediate sibling killing as reported here, it is plausible that they may also serve other roles in microbial competition/predation with other species.

In conclusion, multicellularity offers many advantages to organisms, including increased size to avoid predation, the ability to exploit new environmental niches, and the development of specialized cell functions that allow complex and powerful behaviors. Although many eukaryote species have exploited these advantages, bacterial and archaeal domains have rarely done so. Although the reasons for this remain obscure, one likely hurdle for this evolutionary jump is the evolution of a system whereby individual cells interact with each other in a harmonious and cooperative manner. These behaviors are, at least at face value, at odds with a Darwinian view that individuals (cells) are selfish and seek their own fitness gains. Myxobacteria are attractive model organisms to address this fundamental question because they have evolved a conditional form of multicellularity based on aggregation. One challenge to this strategy is physiological heterogeneity within populations, and here, we showed a case whereby cells resolve this conflict by killing less-fit individuals.

## MATERIALS AND METHODS

### Strains, plasmids, and media.

The bacterial strains, plasmids, and primers used in this study are listed in Tables 1, 2, and 3, respectively. *M. xanthus* was grown in the dark at 33°C in CTT medium (1% Casitone, 10 mM Tris-HCl, pH 7.6, 8 mM MgSO_4_, 1 mM KPO_4_) or in minimal medium A1 (48) or A1 supplemented with 2 mM CaCl_2_. When necessary for selection or induction, media were supplemented with kanamycin (Km; 50 μg/ml), oxytetracycline (Tet; 10 μg/ml), zeocin (Zeo; 50 µg/ml), galactose (Gal; 1%), or IPTG (0.1 mM). The agar (Mo Bio Laboratories) concentration for plates was 1.5%.

**TABLE 1:** Strains used in this study

Strain	Genotype	Comment	Reference
DK1622	Reference *M. xanthus*	WT (Fig. 1A, 2A and B, 3, 4C, 5E)	52
DK8615	DK1622 Δ*pilQ*	Pilus mutant	52
DK6204	DK1622 Δ*mglBA*	Nonmotile prototroph (Fig. 3, 5E)	53
DW1480	DK1622 Δ*traA*	WT Δ*traA* (Fig. 3)	8
DW2412	DK1622 P_IPTG_-tdTomato	DK1622 tdTomato (Fig. 2C and D, 5A to D)	12
DW2602	DK8615 *trpD*::pCR TOPO XL (MXAN_6062)	Tryptophan (Trp) auxotroph (Fig. 2D)	This study
DW2603	DK8615 *hisC*::pCR TOPO XL P_IPTG_-tdTomato	Histidine (His) auxotroph, tdTomato (Fig. 2A and B, 3?, 4C, 5A)	This study
DW2609	DK8615 *hisC*::pCR TOPO XL (MXAN_4228)	HisC auxotroph (Fig. 2C and D)	This study
DW2611	DK8615 *thrC*::pCR TOPO XL (MXAN_3409)	Threonine (Thr) auxotroph (Fig. 2D)	This study
DW2622	DK8615 *gltC*::pCR TOPO 2.1	Nonmotile prototroph (Fig. 3, 5E)	This study
DW2636	DK1622 *argE*::pCR TOPO XL (MXAN_1012)	Arginine (Arg) auxotroph (Fig. 1B, 2D)	This study
DW2650	DK1622 *tsxE*::pCR TOPO 2.1 (MXAN_5572)	*tsxE* mutant (Fig. 5A)	This study
DW2655	DK1622 Δ*tsxEI* (MXAN_5572 to _5571)	*tsxEI* deletion (Fig. 5B to D, 6A)	This study
DW2658	DK1622 Δ*hisC*	*hisC* in-frame deletion; parent of DW2686 (Fig. 1B)	This study
DW2659	DW2650 P_IPTG_-*tsxEI*	*tsxE* mutant expressing *tsxEI* under IPTG-inducible promoter (Fig. 5A)	This study
DW2667	DW2655 P_IPTG_-*tsxI*	*tsxEI* deletion expressing *tsxI* under IPTG-inducible promoter (Fig. 5B)	This study
DW2668	DW2655 P_IPTG_*-*tdTomato	*tsxEI* deletion, tdTomato (Fig. 5C and E)	This study
DW2671	SA5707 P_IPTG_-tdTomato	T6SS deletion, tdTomato (Fig. 5B)	This study
DW2681	DK1622 *tssJ*::pCR TOPO 2.1 (MXAN_4801)	T6SS insertion mutant (Fig. 4C)	This study
DW2686	DW2658 *tsxI*-FLAG	HisC auxotroph expressing *tsxI*-FLAG (Fig. 6A to D)	This study
DW2689	DK1622 *tsxI*-FLAG	WT expressing *tsxI*-FLAG (Fig. 6A, B, and E)	This study
DW2699	DK1622 *trpD*::pCR TOPO TA	Trp auxotroph (Fig. 1B)	This study
DW2700	DK1622 *thrC*::pCR TOPO TA	Thr auxotroph (Fig. 1B)	This study
DW2702	DK6204 *hisC*::pCR TOPO TA P_IPTG_-tdTomato	His auxotroph (nonmotile), tdTomato	This study
DW2703	SA3437 P_IPTG_-*vgrG1* (pVT12)	*vgrG1* deletion expressing *vgrG1*^+^ under IPTG-inducible promoter	This study
DW2704	SA5700 P_IPTG_-*vgrG2* (pVT13)	*vgrG2* deletion expressing *vgrG2*^+^ under IPTG-inducible promoter	This study
SA3437	DK1622 Δ*vgrG1* (MXAN_4800)	T6SS in-frame deletion (Fig. 4C)	54
SA4137	DK1622 ΔMXAN_4807+VipA-GFP (MXAN_4807)	Δ*vipA* expressing VipA-GFP fusion (Fig. 5C)	51
SA5700	DK1622 Δ*vgrG2* (MXAN_5573)	T6SS in-frame deletion (Fig. 4C)	28
SA5701	DK1622 Δ*tagF* (MXAN_4805)	T6SS in-frame deletion (Fig. 4C)	28
SA5707	DK1622 ΔT6SS (MXAN_4800 to _4813)	T6SS deletion (Fig. 4C)	51
SA5712	DK1622 Δ*vgrG1* Δ*vgrG2*	T6SS in-frame deletions (Fig. 4C)	28

**TABLE 2:** Plasmids used in this study

Plasmid	Relevant properties	Source or reference
pCR 2.1 TOPO	Cloning vector, Km^r^	Invitrogen
pCR TOPO XL	Cloning vector, Km^r^ Zeo^r^	Invitrogen
pMR3487	IPTG-inducible promoter, Tc^r^	50
ptdTomato	pMR3487-tdTomato, Tc^r^	Larry Shimkets
pBJ114	Deletion cassette plasmid, Km^r^ Gal^s^	55
pVT1	*hisC* fragment in pCR TOPO XL, Km^r^ Zeo^r^	This study
pVT2	*trp* fragment in pCR TOPO XL, Km^r^ Zeo^r^	This study
pVT3	*argE* fragment in pCR TOPO XL, Km^r^ Zeo^r^	This study
pVT4	*thrC* fragment in pCR TOPO XL, Km^r^ Zeo^r^	This study
pVT5	*gltC* fragment in pCR TOPO XL, Km^r^ Zeo^r^	This study
pVT6	*tsxE* fragment in pCR TOPO 2.1 Km^r^	This study
pVT7	pMR3487-*tsxI*, Tc^r^	This study
pVT8	pMR3487-*tsxEI*, Tc^r^	This study
pVT9	*tssJ* fragment in pCR TOPO 2.1 Km^r^	This study
pVT9	*hisC* in-frame deletion cassette in pBJ114, Km^r^ Gal^s^	This study
pVT10	*tsxEI* deletion cassette in pBJ114, Km^r^ Gal^s^	This study
pVT11	*tsxI* fragment-FLAG in pCR TOPO 2.1 Km^r^	This study
pVT12	pMR3487-*vgrG1* Tc^r^, complementing clone	This study
pVT13	pMR3487-*vgrG2* Tc^r^, complementing clone	This study

**TABLE 3:** Primers used in this study

Primer name^a^	Sequence (5′→3′)
TOPO*hisC*insertion-F	GGTGGACCTGAGTGACAACA
TOPO*hisC*insertion-R	ACTGGTGAGCTTGTAGGGTC
TOPO*thrC*insertion-F	CAGTGAGGGCTGTGACTTTC
TOPO*thrC*insertion-R	AATCACCACCCAGTCCGG
TOPO*argE*insertion-F	GAGTTGGTGGCGATGGACA
TOPO*argE*insertion-R	CAACTGAGACACGCGCTC
TOPO*trpD*insertion-F	CATCATGGGTCTGATGCTCG
TOPO*trpD*insertion-R	AGATTTCATCCAGCCCGTCA
TOPO*gltC*insertion-F	CACCTACGCGCAGAACTTC
TOPO*gltC*insertion-R	GGAACTCGTTGCGGTACTTC
TOPO*tseX*insertion-F	TCATCTACGAGCATCCGGAC
TOPO*tseX*insertion-R	CCCCATCAAAATCCGAGCTG
TOPO*tssJ*insertion-F	GGTGGGGTGTGGTCATCG
TOPO*tssJ*insertion-R	GGTAGCGGAAGACCTCGTC
Xbal-*tsxI*-F	GACGACTCTAGAATGGAGCCCAACAAGGACC
Kpnl-*tsxI*-R	GACGACGGTACCTCACTTCCCGTCTGGCTTG
Xbal-*tsxEI*-F	GACGACTCTAGAATGGAAACGTACGTCGTCAAG
Kpnl-*tsxEI*-R	GACGACGGTACCTCACTTCCCGTCTGGCTTG
Gibson-ΔhisC-Upstream-F	AAACAGCTATGACCATGATTACGCCAAGCTTGAACTGCACACCCACCAC
Gibson-ΔhisC-Upstream-R	ATCGGGACATTCCCTGGCCCTTGATGGAGG
Gibson-ΔhisC-Downstream-F	TCAAGGGCCAGGAATGTCCCGATACGAAGCCC
Gibson-ΔhisC-Downstream-R	ACGACGTTGTAAAACGACGGCCAGTGAATTCAGAGGCGGCTTGTGTCAC
Gibson-Δ*tsxEI*-Upstream-F	AAACAGCTATGACCATGATTACGCCAAGCTTCCTCTACATCACCGAGGCCAT
Gibson-Δ*tsxEI*-Upstream-R	CTTCGCCGGTGCGTCCCCGTCCTTGACGAC
Gibson-Δ*tsxEI*-Downstream-F	AAGGACGGGGACGCACCGGCGAAGCCTCCC
Gibson-Δ*tsxEI*-Downstream-R	ACGACGTTGTAAAACGACGGCCAGTGAATTCGCTCTTTGGTCCGCAGAAGG
TOPO*tsxI-*FLAG-insertion-F	CCCAGTTCGCCATCGTCAAT
TOPO*tsxI-*FLAG-insertion-R	TCACTTGTCGTCGTCGTCCTTGTAGTCCTTCCCGTCTGGCTTGGG
Gibson-*vgrG1*-F	GGATAACAATTAAGGAGGCTATGATCGCGAACGCCGCC
Gibson-*vgrG1*-R	TACGAAGGCGAGCTCGGTACCTAGTTCTCGGAAATCTTGGAGCCC
Gibson-*vgrG2*-F	GGATAACAATTAAGGAGGCTATGGCCGCTCAATTCAATC
Gibson-*vgrG2*-R	TACGAAGGCGAGCTCGGTACTCAGGAACCCTTGTTGATG

a F, forward; R, reverse.

### Plasmid and strain construction.

To create insertion mutations, ~500 bp of the gene of interest was PCR amplified and the amplicon was ligated into pCR2.1 TOPO TA or pCR2.1 TOPO XL vector (Life Technologies) and electroporated into the DH5α *Escherichia coli* strain. Constructs were verified by PCR, restriction analysis, and/or sequencing and were then electroporated into *M. xanthus*.

Markerless deletions were created using a two-step homologous recombination method. Briefly, DNA fragments upstream and downstream of the gene of interest were PCR amplified and cloned into the pBJ114 vector using Gibson assembly (New England Biolabs). Verified constructs were electroporated into *M. xanthus*; transformants were selected for Km^r^ and subsequently counterselected for the loss of galactose resistance (plasmid excision) (49). Markerless deletions were confirmed by PCR using primers flanking the deletion site and by phenotypic analysis.

Inducible gene expression constructs were created by PCR amplification of the gene of interest and ligation of the amplicon into pMR3487 vector (50) downstream of the IPTG-inducible promoter using XbaI and KpnI restriction sites and T4 DNA ligase. Verified constructs were electroporated into *M. xanthus*, and transformants were selected by their ability to grow on medium supplemented with oxytetracycline and confirmed by phenotypic analysis.

A FLAG epitope tag was engineered at the C terminus of TsxI by PCR, and the amplicon was ligated into pCR2.1 TOPO TA. The construct was verified and electroporated into *M. xanthus* as described above.

### Competition experiments.

*M. xanthus* strains were grown in liquid CTT medium overnight and harvested at mid-log growth phase (~3 × 10^8^ CFU/ml). One or both strains were fluorescently labeled with GFP or tdTomato. tdTomato expression was induced by addition of 0.1 mM IPTG to liquid cultures at least 4 h before harvesting of the cells and to solid medium 1 h prior to plating. Cells were washed and resuspended in TPM (CTT without Casitone) to a cell density of ~3 × 10^9^ CFU/ml. Strains were mixed at a 1:1 ratio unless stated otherwise and were transferred to solid medium. After incubation, cells were collected at the indicated times, washed, and then observed on glass slides by phase-contrast and fluorescence microscopy. The competitive index was determined as the change in the ratio between the antagonizing strain (prototroph or toxin producer) and the target strain (auxotroph or toxin-immunity mutant). Typically, 200 to 800 cells were counted per replicate. Alternatively, changes in the strain ratio and cell morphology were observed by direct microscopy on agarose pads with one-half CTT (CTT with 0.5% Casitone) or TPM.

Antagonism was also assessed by determining CFU. Here, competing strains carried different antibiotic resistance markers, and after coincubation, CFU were determined by serial dilutions and plating of strain mixtures on the appropriate selective medium.

Error bars in competition experiments indicate the standard deviation (SD) based on at least three biological replicates performed on different days.

### Western blot analysis.

To determine the relative amounts of TsxI, Hcp, and PilA, cells were incubated under the described conditions and harvested at indicated times. Harvested cells were washed and lysed by sonication in 0.2% CHAPS {3-[(3-cholamidopropyl)-dimethylammonio]-1-propanesulfonate} TPM with protease inhibitor (Complete tablets, Mini EASYpack; Roche). Protein concentration for each sample was determined using a Bradford assay (Bio-Rad), and SDS-PAGE was performed with equal amounts of total protein loaded for each sample. Primary rabbit antibodies used were anti-FLAG (1:5,000; Sigma-Aldrich), anti-Hcp (1:1,000) (51), and anti-PilA (1:7,000) (35). For detection, horseradish peroxidase-conjugated goat anti-rabbit antibody was used (1:15,000; Pierce). Blots were developed using SuperSignal West Pico Plus chemiluminescent substrate (Thermo Scientific).
